# Age at menopause and the prevalence of menopausal symptoms among postmenopausal women in Kabale Municipality in South-Western Uganda

**DOI:** 10.4314/ahs.v25i4.9

**Published:** 2025-12

**Authors:** Leo Odongo, Godwin Turyasingura, Sezalio Masembe, Paula Dhanda, Achim Wöckel, Robert K Silverman, John C Lule

**Affiliations:** 1 Department of Dermatology, Allergology and Venereology, Kabale University School of Medicine, Kabale, Uganda; 2 Department of Obstetrics and Gynaecology, Kabale University School of Medicine, Kabale, Uganda; 3 Department of Obstetrics and Gynaecology, Upstate University Hospital, Syracuse, United States of America; 4 Department of Obstetrics and Gynaecology, Julius-Maximillian University Hospital Würzburg, Würzburg, Germany

**Keywords:** Menopausal symptoms, postmenopause, Kabale Municipality

## Abstract

**Background:**

Postmenopausal symptoms vary between individuals and cultures. There are limited data on postmenopausal symptoms among Ugandan women.

**Objective:**

To determine the age at natural menopause and the prevalence of menopausal symptoms among postmenopausal women in Kabale Municipality, in South-Western Uganda.

**Methods:**

A cross-sectional study on 218 postmenopausal women aged 45 to 65 years from Kabale Municipality to collect data on socio-demographics, reproductive history and menopausal symptoms. Statistical analyses comprised descriptive statistics, Shapiro-Wilk test and two-sample tests of proportions, with significance defined as P < 0.05.

**Results:**

The Median age at menopause was 45 years. 100 % of participants reported experiencing at least one menopausal symptom. Symptoms were most frequently reported in the somato-vegetative domain (98.6%) followed by the psychological domain (95.4%)and least frequently in the urogenital domain (93.6%). The most prevalent individual menopausal symptom was hot flushes (88.1%) and the least prevalent was dyspareunia (14.2%). 73% of participants reported depressive symptoms.

**Conclusions:**

The current study showed the highest prevalence of menopausal symptoms on the African continent. The most prevalent menopausal symptom is hot flushes and the least prevalent is dyspareunia. Urgent psychological support is needed to address the highly prevalent depressive symptoms.

## Introduction

The transition to menopause and the menopausal state involve considerations of both quality of life and disease prevention and management^1^. Menopause is usually diagnosed in retrospect, since confirmation occurs only after a 12-month cessation of menstrual periods^2^. Post-menopause is defined as the stage beginning 12 months after the last menstrual period^3^. Symptoms attributed to menopause vary between individuals and cultures, and have been attributed to general aging, menopause-associated hormonal fluctuations, or socially constructed phenomena^4^. Menopausal symptoms include: those related to menstrual patterns such as shorter cycles, longer cycles and irregular menstrual bleeding; vasomotor symptoms including hot flushes; psychological/cognitive symptoms such as depressive symptoms and poor memory; sexual dysfunction including vaginal dryness, decreased libido, and dyspareunia; somatic symptoms such as headache, palpitations and joint pains; and other symptoms such as dry skin^3^,^5^,^6^,^7^,^8^. The majority of research on menopausal symptoms has been conducted largely on Caucasian women from high-income countries. In Uganda in general and Kabale Municipality in particular, a search of the literature yielded no reported study on the age at natural menopause (ANM) and prevalence of menopausal symptoms. Knowledge of ANM and the prevalence of menopausal symptoms among postmenopausal women in Uganda is important in guiding policy on reproductive health care of the ageing women. The current cross-sectional study therefore set out to determine the ANM and the prevalence of menopausal symptoms among postmenopausal women in Kabale Municipality in South-Western Uganda.

## Methods

### Study Design and participants

A community- based cross-sectional survey was performed on women residing within Kabale Municipality Kabale municipality is situated in South-Western Uganda, and is the only urban municipal council in Kabale district. The municipality is approximately 420 kilometres, by road, southwest of Kampala, Uganda's capital, with 49,186 inhabitants according to the 2014 Uganda population census^20^. The municipality comprises 3 municipal Divisions, 12 wards and 75 cells. Each division has four wards. Included in the study were women aged 45-65 years, able to consent and who had spontaneously stopped menstrual bleeding for 12 consecutive months or more. The women who met the inclusion criteria but were unable to complete the questionnaire were excluded from the study. Of the 221 women approached, 218 (98.6%) agreed to be interviewed and were included in the study. The 218 women interviewed represented 0.4% of the municipality's total inhabitants. Access to the community was obtained through the village Local Council I leaders in conjunction with the village health teams who introduced us to the community and took us to the households containing women whose ages they estimated to be from 45 to 65 years.

We used a consecutive sampling method because the village leaders knew the homes where women within the age range of 45 to 65years were. If in a home there were more than one eligible women, they were all enrolled into the study before moving to the next home.

### Sample Size Calculation

The sample size was 217 participants, determined using the Leslie Kish formula^9^.

The formula is Z 2 pq/d 2

where Z = standard normal deviation at 95% confidence interval = 1.96;
p = prevalence of menopausal symptoms of 83% (0.83) according to a 2015 study from Nigeria^18^;
q = 1 – p =1- 0.83= 0.17;
d = precision limit = 0.05.

### Data Collection

Between February 2019 and August 2019, data were collected using a pre-tested, interviewer-administered questionnaire, which was initially prepared in English and subsequently translated into the Runyankore-Rukiga language. Data collection was done with the aid of three research assistants who were all health workers, aged between 25 and 30 years, all conversant with medical terms in both English and Runyankore-Rukiga languages, were trained to be respectful and sensitive to the participants, and were trained to observe privacy of the patients. The questionnaire contained questions that were formulated by the current study team to obtain data on sociodemographic characteristics and sexual and reproductive history of the participants. Additionally, the questionnaire contained questions which were used to obtain participants' data based on the 11-item Menopause Rating Scale (MRS)^10^. The questions about menopausal symptoms focused on symptoms experienced in the last six months.

The MRS is a self-reported subjective scale that has been used in different international populations and validated in clinical and epidemiological studies on menopause symptoms^11^. The MRS is organized with multiple items on three main areas: (a) Somatic: hot flushes, heart discomfort, sleeping problems, and muscle or joint problems; (b) Psychological: depressive symptoms, irritability, anxiety, and physical or mental exhaustion; and (c) Urogenital: sexual issues, bladder problems, and dryness of the vagina. Patient data were pseudonymized using unique identification numbers and were kept confidential.

### Statistical analysis

We used statistical software STATA version 13. Categorical data were presented as percentages and continuous values were expressed as mean ± standard deviations, or medians and quartile ranges depending on their distribution. For testing the normality of the distributions of quantitative data, the Shapiro-Wilk test was employed. Two-sample tests of proportions were used to test the differences between proportions and we set p <0.05 as level of significance. Participants with missing values for particular analyses were excluded from the analyses.

## Ethical considerations

Ethical approval was obtained from the Research Ethics Committee of Mbarara University of Science and Technology (Reference: MUREC 1/7 13/11-18). Community level approval for the study was sought from the village local council leaders and village health team leaders. All respondents gave written informed consent before participating in the study.

## Results

Sociodemographic characteristics of respondents: The mean age of respondents was 55±5.9 years. The median age of respondents was 55. Most women were married and most of them had attained only primary education (Table 1). The Median age at menopause was 45 years and the range of age at menopause was 36-58 years.

**Table 1:** Socio-demographic features of respondents

Characteristic	n (%)
***Age in years***	
45-50	65(29.8)
51-55	47(21.6)
56-60	63(28.9)
61-65	43(19.7)
***Marital status***	
Single	33(15.1)
Married	84(38.5)
Divorced	12(5.5)
Widowed	89(40.8)
***Level of formal education***	
Tertiary	8(3.7)
Secondary	16(7.3)
Primary	89(40.8)
Church school(club)	15(6.8)
None	90(41.3)

**Note:** Church school refers to learning how to read and write during the catechism classes. Primary education refers to the foundational formal schooling that normally takes seven years in Uganda. Secondary education refers to both ordinary level secondary education and advanced level secondary education which normally lasts four and two years, respectively. Tertiary education refers to education attained at any institution higher than secondary education.

### Prevalence of menopausal symptoms

All respondents (100%, n=218) reported experiencing at least one menopausal symptom, 98.6% (215/218) reported somato-vegetative symptoms, 95.4 % (208/218) had psychological symptoms, and 93.6% (204/218) had urogenital symptoms. As indicated in table 2, the most prevalent menopausal symptom was hot flushes (88.1%, n=192) while the least prevalent individual menopausal symptom was pain during coitus (14.2%, n=31). Also notably, 73% (n=152) of the postmenopausal women reported experiencing depressive symptoms.

**Table 2:** Prevalence of menopausal symptoms experienced in the last six months

MRS domain and individual domain symptoms	Prevalence n (%)
**Somatove getative**	215 (98.6)
Hot flushes	192 (88.1)
Heart discomfort	129 (59.1)
Sleep problems	120(55.1)
Joint and muscle problems	188(86.2)
	
**Psychological**	208(95.4)
Depress symptoms	152(73.1)
Irritability	177(81.2)
Anxiety	170(780)
Physical and mental exhaustion	177(81.2)
	
**Urogenital**	204(93.6)
Vaginal dryness	49(22.5)
Reduced libido	136(62.4)
Dyspareunia	31(14.2)
Urine incontinence	144(66.6)
Urine urgency	136(62.4)

In the somato-vegetative domain of MRS, when we considered joint pain and muscle pain separately, the prevalence of joint pain (80.3%, n=171) was statistically significantly higher than that of muscle pain (72.8%, n=155, p-value < 0.05). Similarly, when we considered, in the psychological domain, physical exhaustion and mental exhaustion as separate components of tiredness, the prevalence of physical exhaustion (73.6%, n=159) was statistically significantly higher than that of mental exhaustion (70.8%, n=153, p-value< 0.05).

## Discussion of results

### The age at natural menopause

The current study found that the the median age at menopause was 45 years and the range of age at menopause was 36-58 years.. Several previous studies have determined mean ANMs for different countries and cultures including 47.7 years in Iran^12^, 48.05 years in Ghana^13^, 48.5 years in Nigeria^14^, 49.30 years in Korean women^15^, 51.0 years in the United Kingdom^16^ and 52.0 years in Australia^17^. These findings are in agreement with the results of previous studies that point to an upward secular trend in ANM with increasing socioeconomic status of countries, with low-income countries having lower mean ANM and high-income countries having relatively higher average ANMs^17^,^18^,^19^. The median ANM of 45years from the current study when compared to the median ANM of 48 years for Iranian, Ghanaian, Nigerian^12^,^13^,^14^ and 49.3 years for Korean women^15^ would still point to the upward secular trend in ANM.

### Prevalence of menopausal symptoms

Whereas we had expected that menopausal symptoms are not a very common health problem in Uganda, the current study found, to our surprise, that all the respondents (100%, n=218) reported experiencing at least one menopausal symptom. This could be one of the few studies on the African continent to report a 100 % prevalence of menopausal symptoms. Among the African studies on menopause we reviewed, only the 2015 Nigerian work by Ibraheem OM et al.^20^ reported overall prevalence of menopausal symptoms experienced in the last one month as 83.8% (n=201). Outside of the African continent, the only study reviewed with prevalence of menopausal women closest to that of the current study was the one carried out in Asian women by Chou MF et al^21^. This study surveyed menopausal symptoms in a convenience sample of 442 Chinese women aged 40-60 years, wherein 98.9% of the women reported experiencing menopausal symptoms. The high prevalence of menopausal symptoms obtained during this current study should prompt a change in policy and practice to ensure active assessment of and treatment for menopausal symptoms. Most surprising and worrying is the high prevalence of depressive symptoms (73%).

Respondents in the current study reported experiencing symptoms mostly in the somato-vegetative domain of MRS (n=215, 98.6%) followed by psychological domain (n=208, 95.4%) and least frequently in the urogenital domain (n=204, 93.6%). These findings are in agreement with the findings from a recent study in Ethiopia by Yisma E and colleagues^22^ wherein the most prevalent types of menopausal symptoms experienced in the last thirty days were in the somatic subscale (65.9%) followed by psychological (46.0%) and urogenital subscale (30.5%). However, the Ethiopian study included participants from a narrower and younger age bracket (30 to 49 years) than in the current study (45 to 65 years) and this would make comparison difficult. The findings from our study and that of Yisma E et al. differed from another African study conducted in Nigeria by Ibraheem OM and colleagues^20^, wherein the major menopausal symptoms described included night sweats 44(18.3%), hot flushes/internal heat were 37 (15.4%), vaginal dryness (81.3%), pain during sexual intercourse (76.7%), loss of sexual urge (69.6%), hair loss (66.3%), urinating more frequently than usual (18.3%), sleep disturbances (16.7%), leaking urine (12.1%), and in stark deviation from our findings only 10.4% reported experiencing depressive symptoms. Considering individual menopausal symptoms in the current study, the most prevalent menopausal symptom was hot flushes (88.1%, n=192) followed by muscle/joint pain (86.2%, n=188), tiredness (81.2%, n=177), irritability (81.2%, n=177), anxiety (78.0%, n=170), depressive symptoms (73.1%, n=152), urine frequency (67.4%, n=147), urine incontinence (66.1%, n=144), reduced libido (62.4%, n=136), and the least prevalent symptom was pain during coitus (14.2%, n=31). Similarly, Yisma E et al [23] also found that the most commonly reported individual symptom was hot flushes (65.9%), which, however, in deviation from our findings was followed by difficulty falling asleep (49.6%), depressive symptoms (46.0%), irritability (45.1%), anxiety (39.8%) and the least prevalent menopause symptoms included heart discomfort (22.1%), bladder problems (26.1%) and sexual problems (27.0%). The differences in prevalence of individual menopausal symptoms observed between our study and that of Yisma E et al. could have arisen from at least two reasons. Firstly, their participants were from a younger age bracket (30-49 years). Secondly, their study involved both perimenopausal and postmenopausal women and this fact also makes comparison of the two studies problematic.

Results differing from the current study are also from an earlier study conducted in Ghana by Kwawukume EY and colleagues, which reported that the major symptoms at menopause were tiredness 79.9%, sleeplessness 71.0%, palpitations 63.7%, weight gain 61.8%, hot flushes 56.5%, and irritability 56.5%^13^. Finally, another research work^24^ from Nigeria, that included a relatively larger number of women (385) and had a wider age range (35-95 years) reported findings differing greatly from our findings in terms of prevalence of menopausal symptoms. The most prevalent menopausal symptoms were loss of libido (92.47%), muscle pain (87.53%), joint pain (85.45%) and tiredness (80.26%). Urinary symptoms had the least prevalence (7.79%). These differing findings, we think, are a result of cultural/ethnic differences in experiencing menopausal symptoms. This is consistent with literature. A study by Monterrosa A et al.^25^ also pointed to ethnic differences in menopausal symptomatology. In their cross-sectional study on 201 healthy Afro-Colombian and 377 healthy non-Afro-Colombian aged 40-59 years, the prevalence of somatic symptoms heart discomfort and muscle and joint problems was reported to be higher among the Afro-Colombian than in non-Afro-Colombian women (38.8% vs. 26.8% and 77.1% vs. 43.5%, respectively); equally, all items of the psychological sub-scale were also found to be higher among black women. This may imply that the black race has more sensitivity to menopausal symptoms. In deed, a 2020 study by Garbose RA et al also reported a high overall prevalence of menopausal symptoms (87%) among African-American women^8^.

In the somato-vegetative MRS domain, when we considered joint pain and muscle pain separately, the current study found, to our surprise, that the prevalence of joint pain (80.3%, n=171) was statistically significantly higher than that of muscle pain (72.8%, n=155) (p-value < 0.05). Similarly, when we considered, in the psychological domain, physical exhaustion and mental exhaustion as separate components of tiredness, the prevalence of physical exhaustion (73.6%, n=159) was statistically significantly higher than that of mental exhaustion (70.8%, n=153) (p-value < 0.05). We think that these differences need to be investigated further. We believe that the differences could have adverse effects on the accuracy and the comparability of results obtained using the 11-item MRS for respondents from different cultural/geographical backgrounds. In particular, we think that lumping muscle pain and joint pain together as well as physical and mental exhaustion together could adversely affect the accuracy, comparaility and the validity of the results of assessment of menopausal symptoms using the MRS, at least for our setting in Uganda. This could imply that the MRS needs to be adjusted to take into consideration this finding in order for the tool to be more suitable for application in Ugandan women.

The current work has the potential to make a contribution to the literature on menopausal symptoms in a relatively poorly studied population in Africa. However, the limitations include: the relatively small sample size, selection bias stemming from disproportionate inclusion of participants across the divisions and wards, the non-random sampling and absence of data obtained from clinical examination of the resondents. Future studies should address these limitations.

## Conclusion and recommendations

Results of this study showed one of the highest prevalence of menopausal symptoms in postmenopausal women. The most prevalent menopausal symptom was hot flush and the least prevalent symptom is pain during coitus.

Specialized psychological support should be urgently provided to the postmenopausal women within Kabale Municipality to address the high prevalence of depressive symptoms. Finally, the MRS needs to be modified and validated for use in Ugandan women.
